# A Huge Abdominal Wall Inflammatory Myofibroblastic Tumor: A Report of a Rare Case and Literature Review

**DOI:** 10.7759/cureus.54795

**Published:** 2024-02-23

**Authors:** Aqeed A Ali, Dalshad H Khurshid, Farman O Shareef, Jeza M Abdul Aziz, Nasreen G Majeed

**Affiliations:** 1 Biomedical Sciences, Komar University of Science and Technology, Sulaymaniyah, IRQ; 2 Surgery, Sulaimani Teaching Hospital, Sulaymaniyah, IRQ; 3 Urology, Sulaimani Teaching Hospital, Sulaymaniyah, IRQ; 4 Medical Laboratory Science, Charmo University, Chamchamal, IRQ; 5 Baxshin Research Center, Baxshin Hospital, Sulaymaniyah, IRQ; 6 Obstetrics and Gynaecology, Baxshin Hospital, Sulaymaniyah, IRQ; 7 Nursing, Azmar Technical and Vocational Institute, Sulaymaniyah, IRQ; 8 Research Center, University of Halabja, Halabja, IRQ

**Keywords:** rare case report, surgical intervention, myofibroblastic spindle cells, abdominal wall mass, inflammatory myofibroblastic tumor

## Abstract

An inflammatory myofibroblastic tumor (IMT), frequently misdiagnosed as a malignant neoplasm, is a rare tumor characterized by the presence of myofibroblastic spindle cells and infiltration of inflammatory cells. In the current study, a 49-year-old female patient with a huge abdominal mass in the left abdominal wall was examined. Diagnostic procedures included blood tests, as well as ultrasound, Doppler, and computed tomography (CT) scans, which revealed the presence of a huge complex multiloculated cystic lesion measuring 30 x 37 x 20 cm. The patient underwent complete excision of the mass. Histopathological examination confirmed the benign nature of the tumor and revealed no evidence of malignancy. A comprehensive review of the available literature shows that the current case is one of the few documented cases. The report concluded by emphasizing the importance of surgical intervention as the primary therapeutic strategy and the crucial role of histopathology in the diagnostic process.

## Introduction

Inflammatory myofibroblastic tumor (IMT), also known as inflammatory (myo)fibroblastic pseudotumor or inflammatory pseudotumor, is a rare benign tumor that is often misidentified as a malignancy or teratoma [1]. According to the current World Health Organization categorization, IMT is classified as a neoplasm that tends to recur locally and is extremely unlikely to spread to other parts of the body. From a histopathological point of view, this disease is characterized by the presence of myofibroblastic spindle cells in association with the infiltration of inflammatory cells, such as plasma cells, lymphocytes, and eosinophils [2]. IMT mainly affects children and young adults, typically aged two to 16 years, and has been observed at various anatomical sites [3]. However, its occurrence in the abdominal wall is extremely rare, even more so in adults. In this report, we present a rare case of a 49-year-old woman with a large IMT in the left abdominal wall that presented as an abdominal wall mass and was treated surgically.

## Case presentation

A 49-year-old woman presented with a large mass on the left side of her abdomen. She first noticed a bulge on the left side of her abdomen five years ago. Although the mass was not initially painful, as it grew over the years, it began to cause discomfort and interfere with her daily activities. In addition, she had no other constitutional symptoms such as fatigue, malaise, loss of appetite, or weight loss. The doctor postponed the surgery owing to concomitant conditions. Thereafter, the condition was ignored by her and her family until it became so severe that it affected her ability to walk and sleep. She was hospitalized for a multidisciplinary approach and preoperative evaluation because she had multiple medical problems. She had already undergone surgery for a toxic goiter and suffered from chronic hypertension, diabetes, and hyperlipidemia. She was also known to have bipolar disorder. She gave birth to six of her seven children vaginally, and the seventh by cesarean section. Apart from the cesarean section, she did not undergo any other gynecological operations, such as a myomectomy.

**Figure 1:**
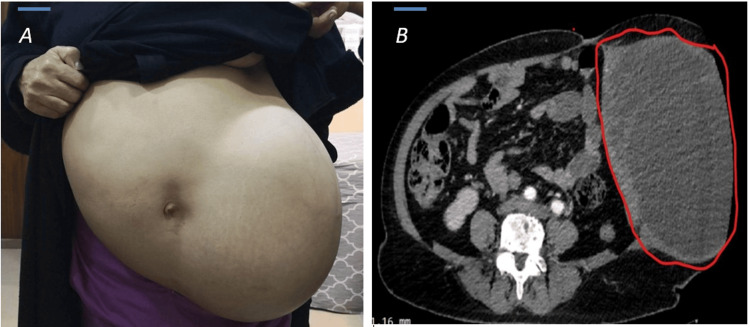
(A) Preoperative clinical photograph showing a large mass occupying the left abdominal wall. (B) Abdominal computed tomography revealed a large (30 x 37 x 20 cm) complex multiloculated cystic lesion on the left side of the abdominal wall.

Upon examination, the patient showed signs of discomfort and was pale. She had an extremely large, mobile, regularly shaped, and non-painful mass filling the entire left side of her abdomen (Figure 1A), in addition to a large left inguinal hernia. The oxygen saturation was 98%, blood pressure was 160/110 mmHg, and heart rate was 83 beats/min. Numerous radiological and blood tests were performed. Complete blood count (CBC) results showed a white blood cell count of 23 10^3^/uL, hemoglobin of 8.1 g/dL, random blood glucose of 220 mg/dL, and glycosylated hemoglobin of 11.3%, indicating that blood glucose was not under control. Renal function tests showed normal urea levels of 25 mg/dL and creatinine of 0.7 mg/dl, and serum electrolytes were normal, except for mild hypokalemia, with sodium at 146.7 mmol/L, potassium at 3.44 mmol/L, chloride at 107.5 mmol/L, and serum calcium at 9.02 mg/dl. The patient also had hyperthyroidism.

Abdominal ultrasound and Doppler ultrasonography showed a 26 × 31 cm heterogeneous hypoechoic soft tissue mass on the left side of the abdominal wall within the muscle layers, without invasion into the intra-abdominal wall cavity. Doppler examination revealed cystic changes, and the mass was hypovascular. Abdominal computed tomography (CT) revealed a large, complex, multilocular cystic lesion measuring 30 x 37 x 20 cm on the left side of the abdomen, suggestive of an abdominal wall defect in the left iliac fossa and groin. The lesion also contained numerous thick septic and solid components (Figure 1B). CT scan of the chest showed a multinodular goiter with retrosternal extension. Echocardiography revealed normal left ventricular function, ejection fraction of 65%, moderate left ventricular hypertension, dilated left atrium, and mild mitral regurgitation. CT angiography was recommended to rule out ischemic heart disease. Coronary CT angiography was performed, and the result was normal. The patient was kept in the ward until her medical conditions such as hypertension, anemia, hyperthyroidism, and blood sugar were controlled by internists, cardiologists, and endocrinologists.

**Figure 2:**
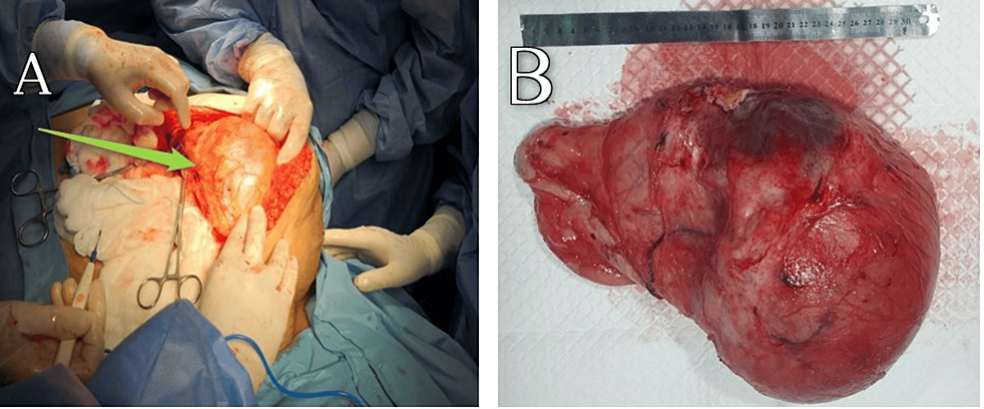
(A) Intraoperative images showing a cystic mass. (B) Postoperative excised abdominal wall mass.

A multidisciplinary team decided to remove the mass under general anesthesia after placing the Foley catheter, sterilizing the abdomen with an iodine solution, and covering it with a sterile compress from the lower chest to the mid-thigh. An incision was made in the midline from the upper to the lower abdomen and a well-defined cystic mass was found under the subcutaneous tissue (Figure 2A). A small incision was made and more than 3 liters of serous-bloody fluid was aspirated from the cyst. The 30 × 20 × 37 cm^3^ cyst was completely excised (the weight was five kilograms), sent for histopathology (Figure 2B), and the wall was sutured in layers. The inguinal hernia repair was also successfully performed after the patient was hospitalized for five days for monitoring and was discharged in good health. The last observation of the patient, four months after surgery, showed no signs of recurrence. Fibrous growing, elongated spindle cells were found without detectable hyperchromasia or cytologic atypia with inconspicuous ovoid nuclei and no significant mitotic activity. Lymphocytes and plasma cells were also found observed. The extracellular matrix ranged from myxoid to collagen with stromal hyalinization, necrosis, and dilated, branched blood vessels with large tumor growth with variable cellularity. Fascicularly growing, elongated spindle cells without recognizable hyperchromasia or cytological atypia with inconspicuous ovoid nuclei and without significant mitotic activity were found. Lymphocytes and plasma cells were also observed. The extracellular matrix ranged from myxoid to collagen with stromal hyalinization, necrosis, and dilated, branched blood vessels with hemorrhage. An area of cystic degeneration was noted. The margins were free of existing lesions. A benign myofibroblastic tumor and malignancy were ruled out, and the result was confirmed by immunohistochemical staining. Cytologically, the abdominal wall mass showed blood and inflammatory cells, but no malignancy.

## Discussion

IMT is a rare soft tissue tumor. The tumors showed abnormal differentiation with marked growth of spindle cells accompanied by inconsistent inflammation. The diagnosis of these lesions is based on their anatomical location, with the lungs being the most commonly affected site. However, Yagci et al. (2010) reported a few cases of IMT occurring outside the lungs [4]. Chen et al. (2007) confirmed that most cases occur in the small intestine, mesentery of the colon, liver, spleen, retroperitoneum, and other gastrointestinal areas [5]. IMTs are rare growths that mostly affect people in the pediatric and young adult groups [6].

The etiology of IMT remains unclear. However, it is thought that various factors such as trauma, inflammation, autoimmune diseases, surgical procedures, and human herpesvirus or Epstein-Barr virus infections may contribute to its development [7]. In most cases, as in our case, no triggering agent was identified. Nevertheless, it was noted that in the present case, seven children were delivered vaginally, with the seventh child being delivered by cesarean section. IMT of the abdominal wall is extremely rare [4]. We performed a comprehensive search on Google Scholar and PubMed using specific search terms: (inflammatory pseudotumor) OR (inflammatory myofibroblastic pseudotumor) OR (inflammatory myofibroblastic tumor) OR (IMT) AND (abdominal wall). Searching PubMed and Google Scholar, we found only six discussed cases with primary IMT of the abdominal wall. Our case is the seventh case of IMT originating from the abdominal wall, and the age at diagnosis varied from six to 50 years, with a mean age of 40.8 years. The incidence of IMT of the abdominal wall was mainly concentrated in middle-aged individuals. The sex distribution showed a remarkable prevalence of males, as shown by the ratio of five males to two females. By comparatively analyzing the size of the mass in our case (30 × 20 × 37 cm^3^) with other cases, it was found that our case had the highest recorded size among primary abdominal wall IMT [4,8-12]. Yagci et al. (2010), Kim et al. (2017), and Pratap et al. (2007) documented that the patients primarily complained of abdominal mass and abdominal pain, which is consistent with the findings of our case studies [4,8,11].

Imaging revealed an indistinct soft tissue mass with heterogeneous enhancement on CT [13]. However, the correct preoperative diagnosis is difficult, and they are difficult to distinguish clinically and radiologically from other soft tissue tumors of the abdominal wall; therefore, an accurate multidisciplinary examination, including clinical examination, histopathology, and radiology, can lead to an accurate diagnosis. In most cases, the final diagnosis is made based on the histopathological findings of either a resected tumor or a needle biopsy. Histologically, IMT is characterized by the presence of spindle-shaped cells and chronic inflammatory cells, including plasma cells, lymphocytes, and scattered histiocytes [14]. All reports to date agree that complete surgical resection is the most important treatment for IMT and that long-term follow-up is necessary to detect local recurrence [15]. All previous reports agree that complete surgical resection is the main treatment for IMT and that long-term follow-up is necessary to detect local recurrence [9]. Rarely does IMT undergo malignant transformation and metastasis, and the risk of distant metastasis is less than 5% [16]. In the present case, immunohistochemistry confirmed a benign IMT and ruled out metastases.

## Conclusions

IMT has also been observed in adults, suggesting that its occurrence in the abdominal wall is rare and not limited to the pediatric population. Histopathology plays a crucial role in distinguishing IMT from other malignant tumors. The main therapeutic approach is comprehensive surgical excision, which may include excision of other affected anatomical components.
